# CASSETTE—clindamycin adjunctive therapy for severe *Staphylococcus aureus* treatment evaluation: study protocol for a randomised controlled trial

**DOI:** 10.1186/s13063-019-3452-y

**Published:** 2019-06-13

**Authors:** Ravindra Dotel, Steven Y. C. Tong, Asha Bowen, Jane N. Nelson, Matthew V. N. O’Sullivan, Anita J. Campbell, Brendan J. McMullan, Philip N. Britton, Joshua R. Francis, Damon P. Eisen, Owen Robinson, Laurens Manning, Joshua S. Davis

**Affiliations:** 1 Centre for Infectious Diseases and Microbiology, Westmead Hospital, University of Sydney, Sydney, Australia; 2 Department of Infectious Diseases, Blacktown Hospital, 18 Blacktown Road, Blacktown, NSW 2148 Australia; 3 Victorian Infectious Disease Service, The Royal Melbourne Hospital–Doherty Department, University of Melbourne, Peter Doherty Institute for Infection and Immunity, Melbourne, Australia; 4 Menzies School of Health Research, Darwin, Australia; 5 Department of Infectious Diseases, Perth Children’s Hospital, Perth, Australia; 6 Wesfarmers Centre for Vaccines and Infectious Diseases, Telethon Kids Institute, University of Western Australia, Perth, Australia; 7 New South Wales Health Pathology, Newcastle, Australia; 8 Department of Immunology and Infectious Diseases, Sydney Children’s Hospital, Sydney, Australia; 9 School of Women’s and Children’s Health, University of New South Wales, Sydney, Australia; 10 National Centre for Infections in Cancer, University of Melbourne, Melbourne, Australia; 11 Department of Infectious Diseases and Microbiology, Children’s Hospital Westmead, Sydney, Australia; 12 Discipline of Child and Adolescent Health, Sydney Medical School and Marie Bashir Institute for Infectious Diseases and Biosecurity, University of Sydney, Sydney, Australia; 13 Department of Paediatrics, Royal Darwin Hospital, Darwin, Australia; 14 Townsville Hospital and Health Service, Townsville, Australia; 15 College of Medicine and Dentistry, James Cook University, Townsville, Australia; 16 Department of Infectious Diseases, Royal Perth Hospital, Perth, Australia; 17 Department of Infectious Diseases, Fiona Stanley Hospital, Perth, Australia; 18 Department of Microbiology, Pathwest Laboratory Medicine, Perth, WA Australia; 19 Antimicrobial Resistance and Infectious Diseases Research Laboratory, School of Veterinary and Life Sciences, Murdoch University, Perth, WA Australia; 20 Faculty of Health and Medical Sciences, University of Western Australia, Perth, Australia; 21 John Hunter Hospital, University of Newcastle, Newcastle, Australia; 22 School of Medicine and Public Health, University of Newcastle, Newcastle, Australia

**Keywords:** *Staphylococcus aureus*, Exotoxins, Prospective studies, Clindamycin, Leukocidins

## Abstract

**Background:**

Exotoxins are important virulence factors in *Staphylococcus aureus*. Clindamycin, a protein synthesis inhibitor antibiotic, is thought to limit exotoxin production and improve outcomes in severe *S. aureus* infections. However, randomised prospective data to support this are lacking.

**Methods:**

An open-label, multicentre, randomised controlled trial (RCT) will compare outcome differences in severe *S. aureus* infection between standard treatment (flucloxacillin/cefazolin in methicillin-susceptible *S. aureus*; and vancomycin/daptomycin in methicillin-resistant *S. aureus*) and standard treatment plus an additional clindamycin given for 7 days. We will include a minimum of 60 participants (both adult and children) in the pilot study. Participants will be enrolled within 72 h of an index culture. Severe infections will include septic shock, necrotising pneumonia, or multifocal and non-contiguous skin and soft tissue/osteoarticular infections. Individuals who are immunosuppressed, moribund, with current severe diarrhoea or *Clostridiodes difficile* infection, pregnant, and those with anaphylaxis to β-lactams or lincosamides will be excluded.

The primary outcomes measure is the number of days alive and free (1 or 0) of systemic inflammatory response syndrome (SIRS) within the first 14 days post randomisation. The secondary outcomes measure will include all-cause mortality at 14, 42, and 90 days, time to resolution of SIRS, proportion with microbiological treatment failure, and rate of change of C-reactive protein over time. Impacts of inducible clindamycin resistance, strain types, methicillin susceptibility, and presence of various exotoxins will also be analysed.

**Discussion:**

This study will assess the effect of adjunctive clindamycin on patient-centred outcomes in severe, toxin-mediated *S. aureus* infections. The pilot study will provide feasibility for a much larger RCT.

**Trial registration:**

Australian New Zealand Clinical Trials Registry, ACTRN12617001416381p. Registered on 6 October 2017.

**Electronic supplementary material:**

The online version of this article (10.1186/s13063-019-3452-y) contains supplementary material, which is available to authorized users.

## Background

*Staphylococcus aureus* is a ubiquitous bacterium, colonising at least 50% of the population (20% persistently and 30% intermittently) [1, 2] and causing a wide range of infections. *S. aureus* bacteraemia (SAB) is associated with a mortality rate of 20–30% in adults [3, 4] and approximately 5% in children [5]. Necrotising pneumonia and severe toxin-mediated infections with *S. aureus*, however, are associated with a much higher mortality [6–8]. This high mortality rate remains despite better understanding of *S. aureus* disease management in recent decades [8–13].

Anti-staphylococcal β-lactams are the drugs of choice for treatment of methicillin-susceptible *S. aureus* (MSSA) infections, whereas vancomycin (or daptomycin) is the drug of choice for methicillin-resistant *S. aureus* (MRSA) infections. Successful source control and early effective antibiotics are the crucial factors for treatment success. Use of a single antimicrobial agent is usually recommended, but two- or three-drug combination therapy is advised in selected cases [12, 13], for instance in prosthetic valve infective endocarditis and prosthetic joint infections. The rationale is either for synergy or biofilm penetration. Additionally, several guidelines [12, 14–16] recommend adding clindamycin to standard therapy in suspected toxin-mediated *S. aureus* infections. These recommendations are based on expert opinions with limited clinical evidence available. Clindamycin is a protein synthesis inhibitor (PSI) antibiotic. Although in vitro animal studies and observational human data suggest a possible benefit of adjunctive clindamycin [17], there are no published or registered randomised controlled trials (RCTs) testing this strategy in *S. aureus* infections.

### *Staphylococcus aureus* toxins

*S. aureus* virulence is in part due to its exotoxins, surface virulence factors, and enzymes [18]. All *S. aureus* strains may produce haemolysins, nucleases, proteases, lipases, hyaluronidase, and collagenase. Many strains also harbour genes for toxic shock syndrome toxin-1 (TSST-1), exfoliative toxins, enterotoxins, and leucocidins [19–23]. In an analysis of 429 *S. aureus* isolates from Germany in 2003 [21] (219 blood isolates and 210 anterior nares isolates, 94% MSSA), 73% of the isolates harboured toxin-related genes. Enterotoxin-G (*seg*), enterotoxin-I (*sei*), and toxic shock syndrome toxin (*tst*) genes were the three most common. In a study by Peacock *et al.* in the UK in 2002 [23], α-haemolysin was near universal in *S. aureus* isolates (both carriage and invasive). Other common toxins were β-haemolysin, δ-haemolysin, γ-haemolysin, enterotoxin-G (*seg*), and enterotoxin-I (*sei*). Twenty-five per cent of carriage isolates and 30% of bacteraemic isolates had the *tst* gene detected.

Panton-Valentine leucocidin (PVL) is a *S. aureus* leukotoxin. Its prevalence varies by population demographics, strain type, and source of infection [24, 25]. PVL-expressing strains have been associated with skin and soft tissue infections (SSTI), which often require surgical intervention [26]. Multi-locus sequence typing (MLST) ST93 *S. aureus* is a virulent strain, and is common in Australia. It carries *lukSF-PV* and hyper-expresses α-toxin [27]. This is true for ST93 MRSA, but MSSA of the same sequence type often co-exists [28].

*S. aureus* virulence is due to multiple determinants, and hence targeting a single factor is unlikely to prove successful in improving disease outcomes [23, 29, 30]. Clindamycin, through its role as a PSI, is expected to limit expression of multiple exotoxins and hence may improve clinical outcomes, but randomised prospective data to support this are lacking.

### Antibiotics which act as protein synthesis inhibitors

Certain antibiotics exert their anti-bacterial activity by selectively blocking prokaryotic ribosomal protein synthesis [31, 32]. The antibiotics targeting the 30S ribosomal subunit (aminoglycosides, tetracyclines, and tigecycline) interfere with the principal function of translating mRNA to peptides. The 50S ribosomal subunit acting antibiotics (clindamycin, linezolid, macrolides, streptogramins, chloramphenicol, and fusidic acid) interfere with peptide bond formation [31]. Specifically, MLS_B_ antibiotics (macrolides, lincosamides, streptogramin B) bind to the narrowest portion of the 50S ribosomal subunit tunnel, which has a regulatory function in peptide synthesis [31].

Of the PSI antibiotics, clindamycin has well-recognised anti-toxin activity. It has shown more consistent toxin suppressive activity than other PSI antibiotics [17] and has been recommended in selected toxin-mediated staphylococcal and streptococcal infections. Linezolid has also been reviewed as an anti-toxin agent [33], but its use is limited by cost, potential for haematological toxicity, and preservation for treatment of resistant Gram-positive organisms and mycobacterial infections.

### Clindamycin compared to β-lactam antibiotics for anti-toxin therapy

β-lactam antibiotics (penicillins, cephalosporins), the drugs of choice for MSSA infections, are less efficacious in high-inoculum infections. Penicillin has been demonstrated as ineffective against bacteria in a stationary phase of growth (i.e. not actively dividing) [34, 35]. Penicillin treatment (including anti-staphylococcal penicillins) may also stimulate toxin production [36–40]. On the other hand, clindamycin is not affected by the phase of growth or inoculum size [29, 35]. It also represses penicillin-induced exotoxin production [40]. Vancomycin and daptomycin appear neither to induce nor inhibit exotoxin production [39, 41].

### Objective

To determine the feasibility of and refine the study design for a future definitive RCT assessing the effect of adjunctive clindamycin on clinical outcomes in children and adults with severe *S. aureus* infections (both MSSA and MRSA).

### Hypothesis

Clindamycin will lead to more rapid resolution of systemic inflammation due to blockade of exotoxin production by *S. aureus*.

## Methods

### Study setting

**Fig. 1:**
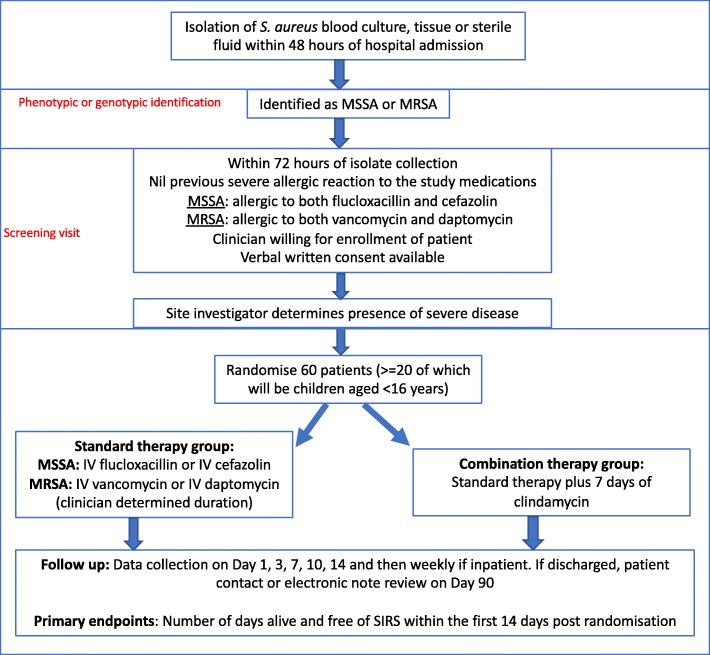
Flowchart overview of the trial design. IV intravenous, MRSA methicillin-resistant *Staphylococcus aureus*, MSSA methicillin-susceptible *Staphylococcus aureus*, SIRS systemic inflammatory response syndrome. SPIRIT checklist (2013) provided in Additional file 2

The CASSETTE study is an investigator-initiated, open-label, parallel-group, superiority, multicentre RCT (Fig. 1). This study will be conducted across 12 hospitals in Australia (see Additional file 1: Table S7). Sites are selected based on: prevalence of *S. aureus* infection (an estimate of at least 10 potentially eligible cases per year for adults and five for children); availability of a committed site principal investigator (PI); and a second person available at each site to assist the PI, either a research nurse, registrar, or physician colleague.

### Inclusion criteria

1. Age—both children and adults will be eligible. Infants must have a corrected age of ≥ 28 days.
2. *S. aureus* (MSSA or MRSA) identified in at least one clinically relevant specimen (including an isolation in a polymicrobial culture when the other isolates are thought to be non-significant).

A “clinically relevant specimen” refers to any specimen where the site PI judges the isolate to be contributing to the patient’s clinical syndrome. This does not have to be a sterile site. These specimens may include blood, fluids from other normally sterile sites (such as joint fluid or cerebrospinal fluid), pus, tissue, or bone obtained surgically. The site PI may additionally make a clinical judgement on whether other specimens from non-sterile sample types yielding *S. aureus*, such as sputum and superficial swabs, are considered clinically relevant.

3. Ability to be randomised within 72 h of the collection of the index culture.

The index culture is the first clinically relevant specimen collected both after the participant has arrived at a hospital and after the onset of systemic symptoms. If more than one specimen is collected within the same 4-h period, the most clinically significant specimen will count as the index specimen (sterile site isolates are prioritised over others). Randomisation will be performed using a web-based system (REDCap).

Antimicrobial therapy will be clinician determined prior to randomisation. This may include clindamycin and a combination antibiotic that may include coverage for both MSSA and MRSA. The pre-randomisation treatments will be recorded and adjusted for in the analysis.

4. Probability of remaining as an inpatient of the study site hospital for at least 7 days following randomisation (or accessible for follow-up by the site PI, e.g. hospital in the home).
5. Index culture drawn no later than 48 h after hospital admission.
6. Severe disease: evidence of ≥ 2 systemic inflammatory response syndrome (SIRS) criteria within 48 h prior to randomisation and at least one of the following three conditions.
  6.1. Septic shock (including Staphylococcal toxic shock syndrome).

In adults, septic shock is defined as:

i. Mean arterial pressure < 70 mmHg or systolic blood pressure < 90 mmHg despite at least 3 L of fluid administration; or
ii. the need for intravenous vasopressors to maintain organ perfusion (i.e. any receipt of adrenaline, noradrenaline, dopamine, dobutamine, vasopressin, or terlipressin).

In children, septic shock is defined as sepsis with cardiovascular dysfunction. Sepsis is SIRS in a setting of suspected or proven infection. Cardiovascular dysfunction is defined by the International Paediatric Sepsis Consensus Conference [42] as follows—despite administration of isotonic intravenous fluid bolus ≥40 ml/kg in 1 h:

i. decrease in blood pressure (BP) (hypotension) < 5th percentile for age or systolic BP < 2 SD below normal for age (Table 1); or
ii. need for vasoactive drug to maintain BP in normal range (dopamine > 5 μg/kg/min or dobutamine, epinephrine, or norepinephrine at any dose); or
iii. two of the following:
  - unexplained metabolic acidosis: base deficit > 5.0 mEq/L;
  - increased arterial lactate > 2 times upper limit of normal;
  - oliguria: urine output < 0.5 ml/kg/h;
  - prolonged capillary refill: > 5 s; and
  - core to peripheral temperature gap > 3 °C.

6.2. Necrotising lung/pleural space infection

**Table 1:** Age-appropriate criteria for paediatric systemic inflammatory response syndrome

Age group	Heart rate (beats/min)		Respiratory rate (breaths/min)	Leucocyte count × 10^3^/mm^3^	Systolic BP (mmHg)
	Tachycardia	Bradycardia			
1 month–1 year	> 180	< 90	> 60	> 17.5 or < 5	< 100
2–5 years	> 140	NA	> 40	> 15.5 or < 6	< 94
6–12 years	> 130	NA	> 20	> 13.5 or < 4.5	< 105
13–15 years	> 90	NA	> 20	> 11 or < 4.5	< 117

The values are the 5th or the 95th percentile for age

Modified from Goldstein et al.’s paediatric sepsis consensus report [42]

*BP* blood pressure, *NA* not applicable

Either:

i. an exudative pleural fluid aspirate and has Gram-positive cocci in clusters on Gram stain (in the presence of MSSA or MRSA from another clinically relevant specimen) or has grown *S. aureus* from the pleural fluid; or
ii. pneumonia which is:
  - multifocal (within the lung); and
  - *S. aureus* grown from any of sputum, endotracheal aspirate, bronchial lavage, blood, or pleural fluid.

The diagnosis of necrotising pneumonia may be aided by computerised tomography (CT) scan, if available.

6.3. Complicated skin/soft tissue/osteoarticular infection which is multifocal and non-contiguous
  This includes pyomyositis, septic arthritis, osteomyelitis, skin or soft tissue abscess, or carbuncle.

Non-contiguous multifocal means it is at more than one anatomical site, which is likely to be due to systemic spread rather than direct extension. For example, osteomyelitis of the distal tibia with septic arthritis of the ankle joint does not qualify, as it is contiguous. Lumbar vertebral osteomyelitis with direct extension into the psoas muscle does not qualify. Humeral osteomyelitis with psoas abscess does qualify.

### Exclusion criteria

1. Previous severe allergic reaction to both flucloxacillin and cefazolin (for MSSA), or to both vancomycin and daptomycin (for MRSA), or to lincosamides.
2. Previous participation in the trial.
3. Known pregnancy.
4. Currently receiving a lincosamide or other potentially anti-toxin antibiotic which cannot be ceased or substituted (listed in the earlier section “Antibiotics which act as protein synthesis inhibitors”).
5. Participant’s primary clinician unwilling to enrol patient.
6. Moribund (expected to die in next 24 h with or without treatment).
7. Treatment limitations which preclude the use of antibiotics.
8. Significant immunosuppression (prednisolone > 0.5 mg/kg/day for ≥14 days in the last 30 days, other immunosuppressive medication, known HIV with CD4 count < 200, congenital immunodeficiency).
9. Necrotising fasciitis.
10. *Clostridiodes difficile*-associated diarrhoea or severe diarrhoea (> 6 stools per day or clinician-determined severe diarrhoea in children) from any cause.

### Interventions

#### Control group

The control group will receive “standard therapy”, also called “backbone therapy”. This will consist of IV flucloxacillin 50 mg/kg/dose up to 2 g every 4–6 h given intermittently or via 24-h infusion for MSSA infections. Flucloxacillin can be substituted with IV cefazolin 50 mg/kg/dose up to 2 g every 6–8 h if there is a history of minor allergic reaction to penicillin, defined as rash or unclear allergic reaction, but not anaphylaxis or angioedema, or at the site PI’s discretion. The standard therapy for MRSA infection will consist of IV vancomycin with a loading dose of 25 mg/kg in adults (clinician discretion regarding loading dose in children < 18 years) followed by maintenance dose of 15–20 mg/kg every 12 h (for adults) or 15 mg/kg/dose every 6 h (for children) with subsequent adjustment to maintain trough levels at 15–20 mg/dl, which may require an infusion at clinician discretion or IV daptomycin 6–10 mg/kg/day. Paediatric daptomycin dosing guidance [43]: for 1–6 years old, 12 mg/kg/dose every 24 h; for 7–11 years old, 9 mg/kg/dose every 24 h; and for 12–17 years old, 7 mg/kg/dose every 24 h. The dose of backbone therapy will be adjusted for renal function (Additional file 1: Tables S1–S5).

The duration of standard therapy is clinician determined but should be in line with recommendations in the current version of Therapeutic Guidelines (TG) Antibiotic [14] in adults and with expert consensus in children. The choice between vancomycin and daptomycin, and flucloxacillin and cefazolin is clinician determined, and may be based on local practice or the minimum inhibitory concentration (MIC) of the MRSA isolate against vancomycin.

The usual recommended duration of a standard treatment of *S. aureus* sepsis in adults is 14–42 days. In children, early switch (after 7–14 days of IV therapy) to oral therapy is practised commonly and is permitted in this trial.

#### Combination therapy group

All participants assigned to the combination group will receive standard therapy as above. In addition, they will also receive open-label clindamycin (10 mg/kg/dose up to 600 mg QID IV for both adult and children) for 7 days from randomisation (day 1 being the day of randomisation). The intravenous route for clindamycin is recommended but not mandated for the entire duration of therapy (7 days). If a switch to oral therapy is thought to be necessary and appropriate by the site PI, transition to oral clindamycin 450 mg TDS for adults or 10 mg/kg/dose PO TDS (maximum 450 mg) for children is recommended.

#### Criteria for discontinuing or modifying allocated interventions

- Adjustment for renal impairment.

Dosage adjustment for the standard therapy medication is as per the recommendations in TG [14] and antimicrobial dosing in renal impairment guidelines of the Children’s Hospital [44] at Westmead, Sydney, Australia (Additional file 1: Tables S1–S5; also includes vancomycin therapeutic drug monitoring). Continuous infusion of the backbone antibiotics (flucloxacillin/cefazolin/vancomycin) during inpatient admission is not routine practice in Australia; however, such use will not constitute a protocol violation. Clindamycin (IV or PO) does not need dose modification in renal impairment.

b. Change of primary standard therapy after randomisation.

The change of primary standard therapy between flucloxacillin and cefazolin (for MSSA) or between vancomycin and daptomycin (for MRSA) is permitted at the discretion of treating clinicians, or if there are issues with supply or stock of these antimicrobials. Any unnecessary changes will be discouraged. Clinicians may change the backbone therapy if the patient develops an adverse drug reaction, such as rash with flucloxacillin or vancomycin, or raised creatinine kinase with daptomycin, or if the vancomycin MIC of the MRSA isolate is found to be ≥ 2 μg/ml, or in cases of persistent bacteraemia whilst receiving vancomycin.

The change within 7 days of randomisation will still be counted as β-lactam therapy and will not violate per-protocol analysis. However, it will affect the subgroup analysis (flucloxacillin vs cefazolin; vancomycin vs daptomycin). Participants who receive the majority of one or the other drugs in the 7 days will be analysed in the respective groups.

c. Clindamycin use after day 7 post randomisation.

The use of clindamycin after the completion of 7 days of randomisation will be discouraged for up to 90 days post randomisation. The treating team will be advised to use an alternative to clindamycin if such treatment is necessary.

#### Strategies to improve adherence to the protocol, and any procedures for monitoring adherence

- Training of site investigators.

Each site will have a PI who will be trained in the study protocol, standard operating procedures, and their reporting requirements. The project manager will have regular contact (email or phone) with all enrolling site PIs.

b. Documentation in patient’s medical records.

On day 1 of randomisation, the patient’s enrolment in the CASSETTE trial will be documented in the medical record, and the treating team will be made aware of the recruitment. Study information will be placed in the patient’s folder. The PI or their associates (research nurse or registrar) will check drug charts (paper and/or electronic) daily (except weekends and public holidays) during the period of the intervention. Charts will be reviewed before weekends and public holidays to ensure appropriate doses are prescribed for the period, and to check when clindamycin requires ceasing (i.e. will complete 7 days). Follow-up will be done on the first working day.

c. Monitoring.

As this pilot study is determining feasibility as well as refining assumptions and study design in preparation for a definitive RCT, we intend to utilise off-site risk-based monitoring to be completed by the central coordinating office. The web-based database will have logic checks included in the design to avoid data-entry errors. The central coordinator will also monitor data entry at each site for completeness and data checks will be regularly conducted to ensure protocol compliance.

#### Relevant concomitant care and interventions that are permitted or prohibited during the trial

Apart from the use of clindamycin, the patient’s management will be at the discretion of the caring team in consultation with the site PI. Procedures undertaken and directly relevant to the study will be recorded (e.g. debridement of infected necrotic tissue, washout of an infected joint, or use of intravenous immunoglobulin). Actions that clearly violate the study protocol, such as early cessation of clindamycin or use of an additional PSI antibiotic, will be discouraged. Site investigators will actively recommend therapeutic drug monitoring or dose adjustment for renal failure per protocol as necessary.

### Outcomes

#### Primary outcome

The primary outcome is the number of days alive and free of SIRS within the first 14 days post randomisation. “Free of SIRS” is defined as meeting < 2 SIRS (i.e. 1 or 0) criteria simultaneously. The day of randomisation is counted as day 1 (not day 0). We used SIRS criteria as they are routinely collected and are a more conservative measure of patient recovery. Also, this was thought to be a single measure of sepsis that could be used in both children and adults.

##### SIRS criteria in adults [45]

1. Abnormal body temperature (< 36 or 38 °C).
2. Tachypnoea or mechanical ventilation (RR > 20 breaths/min in an adult, age dependent in children).
3. Tachycardia (HR > 90 beats/min in an adult, age dependent in children).
4. Abnormal leucocyte count, > 12 × 10^9^/L or < 4 × 10^9^/L (using last observation carried forward from any FBC).

##### SIRS criteria in children (Table 1)

SIRS is defined as the presence of at least two of the following four criteria, one of which must be abnormal temperature or leucocyte count. The following are modified from the NSW Clinical Excellence Commission Sepsis Kills guidelines [46] and the International Paediatric Sepsis Consensus Conference [42] held in 2002. Heart rate, respiratory rate, and temperature will be recorded at least daily for the first 7 days, and after this time will be recorded at least daily as long as the patient remains in hospital or hospital in the home (HITH).

1. Abnormal body temperature of < 36 or > 38 °C.
2. Tachycardia or bradycardia (mean heart rate) that is otherwise unexplained and persists over at least 30 min.
3. Tachypnoea (see Table 1) or mechanical ventilation not related to underlying neuromuscular disease or the receipt of general anaesthesia.
4. Abnormal leucocyte count, or > 10% circulating immature neutrophils (“band forms”).

SIRS is considered present if ≥ 2 criteria were met at the same time on any given calendar day. For example: an adult had a heart rate of 95 beats/min at 9 a.m. but no other criteria at this time. She then had a heart rate < 90 beats/min for the rest of the day. At 3 p.m. she had a respiratory rate of 22 breaths/min for 1 h which also then normalised. Although she had two SIRS criteria on the same calendar day, they were not simultaneous and hence she was deemed to be free of SIRS on that day.

If the patient has been discharged from both hospital and HITH before day 14, then SIRS will be assumed to have resolved at the time of discharge if it had not already done so.

White blood counts will be measured on days 1, 3, 5, 7, and 14. There is a ± 1-day window if the WBC is not collected on the stipulated days, with preference given to the preceding day’s results. If more than one WBC is measured on the same day, then the most abnormal results will be recorded.

### Secondary outcome measures

1. All-cause mortality at 14, 42, and 90 days.
2. Time to resolution of SIRS (number of days until the patient meets < 2 simultaneous SIRS criteria on a calendar day).
3. Proportion with microbiological relapse (positive blood culture for MSSA or MRSA at least 72 h after a preceding negative culture).
4. Proportion with microbiological treatment failure (positive sterile site culture for MSSA or MRSA at least 14 days after randomisation).
5. Number of surgical procedures performed for the purposes of *S. aureus* infection source control.
6. Duration of intravenous antibiotic treatment.
7. *C. difficile*-associated diarrhoea (3 or more loose stools per day along with a positive laboratory test for *C. difficile* toxin).
8. All cause diarrhoea (3 or more loose stools per day).
9. Slope of CRP curve days 1–14 (i.e. rate of change of CRP over time).

### Participant timeline (Table 2)

#### Eligibility screening

We will identify potential patients as those who meet the two main inclusion criteria of: *S. aureus* (MSSA and MRSA) identified in a clinically relevant specimen; and clinically defined severe disease (septic shock, necrotising lung/pleural space infection, complicated multifocal skin or soft tissue, or osteoarticular infection). All such identified patients will be screened and assessed for potential enrolment and randomisation. The identification of such participants will typically be through notification by microbiology staff (e.g. when blood cultures are called through and clinical information obtained from the treating clinician) or other medical staff (e.g. infectious diseases, paediatric, ICU). Identification of *S. aureus* as MSSA or MRSA can be by any acceptable methods, such as nucleic acid amplification of *fem/nuc, mec* genes, or phenotypic susceptibility methods.

**Table 2:** Schedule of visits, data collection, and follow-up

Timepoint	Day 1	Day 2	Day 3	Day 4	Day 5	Day 6	Day 7	Da y 10	Day 14	Day 15–89	Day 90
Enrolment											
Eligibility screen	X										
Informed consent	X										
Demographic details	X										
Clinical details	X										
Randomisation	X										
Interventions											
Standard therapy (flucloxacillin/cefazolin or vancomycin/daptomycin)	X	X	X	X	X	X	X	X	X^a^	X^a^	
Clindamycin (if in combination group)	X	X	X	X	X	X	X				
Assessments											
Blood cultures			X		X^b^		X^b^				
FBC, EUC, LFTs, CRP	X		X		X		X		X		
Check SIRS score	X	X	X	X	X	X	X	X	X		
Check vital observations	X		X				X	X	X		
Clinical progress assessment	X		X				X	X	X	Weekly if inpatient	X
Vital status			X				X	X	X	X	X
Additional data											X

*CRP* C-reactive protein, *EUC e*lectrolytes, urea, and creatinine, *FBC* full blood count, *IV* intravenous, *LFT* liver function tests, *SIRS* systemic inflammatory response syndrome

^a^The usual duration of IV treatment for Staphylococcus aureus bloodstream infection is 14–42 days in adults and 7–14 days in children; 4–6 weeks of IV therapy may be necessary in children with infective endocarditis

^b^If day 3 blood culture is positive, repeat every 48–72 h until negative

#### Informed consent

The PI or their delegate will approach an eligible patient or their next of kin (NOK) for a discussion on participation in the trial. Written information will also be provided. An interpreter will be used if necessary. Informed consent from a NOK will only be used in jurisdictions where there is legislative approval to do so, and where the site has research governance approval in place.

#### Paediatric-specific consent issues

All participants under the age of 18 years will have consent sought from a parent/guardian. Children and adolescents will have the study explained to them using appropriate language. Where deemed appropriate, the adolescent will be asked to co-sign the parent/guardian consent form. If an adolescent does not want to participate in the study, this would be considered a refusal and they will be considered ineligible.

For Aboriginal and Torres Strait Islander children, informed consent can be given by a parent or caregiver who is recognised under Aboriginal customary law or Aboriginal tradition as having decision-making rights over the child’s health care needs and who assumes day-to-day parental responsibilities.

If consenting parents are under the age of 18 years, consent will be sought from the child’s grandparent with a parent signing as a witness.

#### Randomisation and blinding

Patients will be randomised using a module in the web-based study database. The randomisation list will be generated and held by an independent statistician. Randomisation will be stratified by age (child versus adult) in a 1:1 ratio, in permuted blocks of variable size. Randomisation will not be stratified by site, as an average site is only likely to randomise 5–10 patients and hence the strata will be too small. We will not stratify by MSSA/MRSA status for similar reasons—the strata will be too small, and the study is designed as a pilot study with the aim of assessing for feasibility.

Blinding is not relevant as this will be an open-label trial.

#### Days 2–90

The PI (or delegate) will visit the patient, review medical documentations, or contact the treating team in person or by phone daily for the first 7 days of randomisation to ensure compliance with the protocol and that the recommended tests are ordered. Case report forms (CRFs) with details on illness severity scores, clinical progress, and blood results will be completed daily. These details can be retrospectively collected up to 72 h after the intended day, allowing for weekends and public holidays. Standard operating procedures will contain step-by-step details on how to recruit patients and collect data.

#### Endpoint assessment

A panel blinded to treatment allocation will determine the primary endpoint. This will consist of three clinicians, each with expertise in infectious diseases, paediatrics, or critical care. The critical care clinician will chair to overcome discrepancies in the decision-making between the three decision-makers.

The panel will be given an extract of the database which contains all information needed to determine the number of days alive and free of SIRS over the first 14 days but will not be given any information about the use of clindamycin.

If needed, the panel may request further information (e.g. medical record or observation chart extracts with identifying information redacted) for any particular patient.

### Discontinuation/withdrawal of participants from trial treatment

Participants or NOK can voluntarily withdraw from study at any time if they wish to. Investigators may also discontinue a participant from the study if deemed appropriate at any time. Participants do not necessarily need to provide explanations for their withdrawal. If withdrawn by the site investigator, reasons for discontinuation will be recorded in CRFs. Participants will not be withdrawn from the study due to suspected adverse reactions of study treatment, unless the participant or their treating clinician requests discontinuation. If the participant or NOK withdraws consent to participate in the study and also withdraws consent for collection of future information, no further evaluations will be performed, and no additional data will be collected. The investigators may retain and continue to use any data collected before such withdrawal of consent. All withdrawal/discontinuation will be discussed with the coordinating investigators.

To avoid missing data, and for use in intention-to-treat analysis, all attempts will be made to collect information at day 90 for participants who discharge early, transfer to another facility, discharge against medical advice, abscond, or are lost to follow-up.

### Sample size considerations

Sixty patients will be enrolled, at least 20 of whom are aged < 16 years. As this is a pilot study, the sample size is based on the number achievable, the number needed to determine feasibility, and to refine assumptions and study design.

### Data management

#### Data collection

All initial data collection can either be in paper format (with subsequent entry into the electronic database) or directly entered into the electronic database. For database entry, each variable will have a validation range to minimise entry errors. The coordinating centre will check data for consistency and will seek any missing data with help of the PI.

During randomisation, each participant will be given a unique identifier number. This number and the hospital medical record number will be recorded in each CRF. These two numbers will be cross-checked each time data entry is performed in CRFs to ensure correct documentation. The date, time, and name of the person performing data entry for each entry in the CRF will be mandatory. Any changes made in the CRF will be timed, dated, and signed and the reasons for changes documented. CRFs will not contain participant identifying details such as address, initials, or date of birth, but will contain age at recruitment.

Clinical details will be obtained from medical records (paper and/or electronic), bedside charts, medication charts, pathology results, correspondence notes, and telephone contact with the patient or their NOK. Clarification will be made with the treating team or the participants’ general practitioners if necessary.

#### Data entry and storage

Data collected in paper CRFs will be entered onto a purpose-built secure web-based database. This will be done at each site by the PI or delegate. Paper CRFs will be securely stored at each site. Data will be retained for at least 10 years after study completion.

### Statistical methods

Data reporting will follow CONSORT guidelines for reporting of RCTs.

Continuous variables will be analysed using the Mann–Whitney *U* test or Student’s *t* test as appropriate. Proportions will be analysed by Fisher’s exact test or chi-squared tests as appropriate. The 95% confidence interval will be reported for absolute difference in proportions. All-cause mortality will be shown with Kaplan–Meier graphs.

The primary analysis of both primary and secondary endpoints will be according to modified intention-to-treat principles (all participants with data available for the endpoint will be analysed according to the treatment allocation, regardless of what treatment they received).

Secondary per-protocol analysis of all endpoints will be conducted. The per-protocol population is defined as: the combination group that received at least 75% of clindamycin dose; the standard treatment group that received ≤ 1 defined daily dose of clindamycin in the week preceding randomisation; and those with data available for at least 90 days of follow-up.

There will be no planned interim efficacy analyses.

#### Pre-specified subgroup analyses

The number of subgroup analyses will be limited due to the small sample size:

i) Main treatment was flucloxacillin vs cefazolin (for the MSSA cohort). Recent data suggest better outcomes for SAB with cefazolin versus nafcillin/oxacillin [47], although it is unclear whether this is true, and the effect size is likely to be small.
ii) Those who received > 24 h of lincosamides or other anti-toxin antibiotics (as defined in the eligibility criteria) in the 7 days prior to randomisation, compared with those who did not. Recent receipt of lincosamides is likely to dilute the effect of the randomised intervention.
iii) Children vs adults. The distribution of outcomes is likely to be different in children from that in adults, although the effect of the intervention should not differ.
iv) MSSA vs MRSA infection. MRSA isolates may have higher rates of clindamycin resistance or may involve patients within certain risk groups (e.g. intravenous drug users, haemodialysis patients, or recent hospitalisation).

### Data safety and monitoring board

An independent data safety and monitoring board (DSMB) will be established to review the progress of the study and monitor adherence to the protocol, participant recruitment, outcomes, complications, and other issues related to participant safety. They will also monitor the assumptions underlying sample size calculations for the study and alert the investigators if they see substantial departures from the study protocol as the data accumulate.

The DSMB will be composed of experts in infectious diseases (adult representative and paediatric representative) and biostatistics, and an intensivist. The DSMB members will all be independent of the investigators (none of them will be chief investigators or site PIs).

### Safety aspects of the trial

#### Adverse events and adverse reaction (solicited and unsolicited)

Clindamycin is registered in Australia for therapeutic use. Clindamycin has a good safety profile but has some recognised adverse effects [48]. Antibiotic-associated diarrhoea and *C. difficile* diarrhoea are the most frequently encountered clinical adverse event with clindamycin. Rash, blood dyscrasias, and raised liver enzymes could be difficult to attribute to clindamycin, especially since the trial participants will also be receiving β-lactams and have a severe infection. Nonetheless, these and any other potential adverse events in both standard therapy and combination therapy groups will be monitored and recorded in CRFs*.* Clindamycin can be ceased if the patient develops *C. difficile* diarrhoea, severe non-*C. difficile* diarrhoea, or other serious adverse events.

Vancomycin adverse events include phlebitis, nephrotoxicity, rash, and red-man syndrome (if infused rapidly). Daptomycin adverse events include headache, rash, infection site reactions, increased creatinine kinase, increased liver enzymes, and hypotension. Less common adverse effects of vancomycin include immune-mediated thrombocytopenia and ototoxicity, and those of daptomycin include eosinophilic pneumonia [48].

Adverse events will be graded as follows (Additional file 1: Table S6) [49]:

- Grade 1: mild symptoms. Causes nil or minimal interference with usual social and functional activities. No intervention indicated.
- Grade 2: moderate symptoms. Interferes with but does not limit usual social and functional activities. Intervention is indicated.
- Grade 3: severe symptoms. Inability to perform usual social and functional activities. Intervention or hospitalisation is needed.
- Grade 4: potentially life-threatening symptoms. Inability to perform basic self-care functions. Intervention is indicated to prevent permanent impairment, persistent disability, or death.
- Grade 5: death related to adverse event.

#### Serious adverse events

Serious adverse events (SAEs) are an undesirable experience as a result of the intervention that:

- ◦ results in death;
- ◦ is life-threatening;
- ◦ results in hospitalisation (initial or prolongation);
- ◦ results in disability or permanent damage; or
- ◦ is a medically important event or reaction.

All SAEs that are considered related (possible, probable, or definite) to any of the study drugs (backbone and combination therapy) which met one or more the above definitions require reporting on the SAE form. If the SAE is attributable to disease progression then this does not require expedited reporting in this trial, even when death is the outcome. The site PI is required to report any SAEs that occur at their site to the approving ethics committee in accordance with local guidelines; in addition, the site PI must adhere to any local institutional reporting requirements. Safety reporting will be in line with the National Health and Medical Research Council [50]. Guidance: Safety monitoring and reporting in clinical trials involving therapeutic goods. Canberra: National Health and Medical Research Council.

#### Suspected unexpected serious adverse reaction

If the SAE is considered unexpected (not listed in approved product information and not attributable to disease progression) and related (possible, probable, or definite) to the investigational drug (clindamycin), it meets the definition of a suspected unexpected serious adverse reaction (SUSAR). All SUSARs must be entered on the SAE CRF and reported to the coordinating investigators (CIs) or delegate within 24 h from the time of the site study team becoming aware of it. If the SUSAR is fatal or life-threatening, the PI will report to the TGA within 7 calendar days of becoming aware and any follow-up reports will be submitted within a further 8 calendar days; all other SUSARs will be reported to the Therapeutic Goods Administration (TGA) within 15 calendar days of the PI becoming aware.

The site PI is required to report any SUSARs that occur at their site to the approving ethics committee in accordance with local guidelines; in addition, the site PI must adhere to any local institutional reporting requirements. Safety reporting will be in line with the NHMRC Guidance: Safety Monitoring and Reporting in Clinical Trials Involving Therapeutic Goods [50].

The relation of adverse event to the treatment will be defined as not related, unlikely, possible, probable, and definite [51].

### Ethical considerations

The trial will be conducted in line with the Declaration of Helsinki. Written informed consent will be obtained from all study participants prior to any study procedure. Approval from a lead HREC and site-specific approval will be finalised at each site before the first patient is enrolled at that site. There will be two lead HRECs: one for the NT site (Menzies and NT Department of Health EC00153), and one for the remaining sites as per the National Mutual Acceptance scheme (Hunter New England EC00403).

#### Dissemination policy

The chief investigators (JSD, SYCT, AB) will have access to the study dataset. The results of this study will be submitted to peer-reviewed journals for publication regardless of the results. They will also be presented at national and/or international scientific meetings. Authorship of the main paper will be all PIs, “on behalf of the CASSETTE study group, the ASID CRN and Australia and New Zealand Paediatric Infectious Diseases Group (ANZPID)”. The CASSETTE Study Group will consist of all CIs and all site PIs, as well as up to two co-investigators per site if relevant.

## Discussion

Although animal studies and observational reports support the concept of effective *S. aureus* exotoxin suppression with clindamycin [17], adequate supporting clinical evidence is lacking. The proposed study is an open-label, pilot, randomised controlled trial to determine whether 7 days of clindamycin in combination with standard therapy will lead to a faster resolution of systemic inflammation than standard therapy alone in adults and children with severe *S. aureus* infection.

It is unclear whether the anti-toxin effect of clindamycin is retained in *S. aureus* strains that are resistant to it. This may be a limiting factor for our study design. Fortunately, most of the MSSA isolates in Australia have low rates of constitutive resistance against clindamycin (0.8–2.4% of blood culture isolates) [52–54]. Clindamycin resistance is, however, more common in MRSA isolates (16.5–21%) [52, 53]. Inducible resistance to clindamycin due to the presence of a mutation in the *erm* gene is much more commonly observed as a potential issue in isolates resistant to erythromycin (~ 50% of MRSA isolates from blood culture are erythromycin resistant) [55], although not all erythromycin-resistant strains are resistant to clindamycin. Resistance to macrolides due to efflux pumps does not create resistance to lincosamides (clindamycin and lincomycin). In the peptide tunnel of the 50S ribosome subunit, each of MLS_B_ antibiotics has its own interaction sites. Methylation at the common interaction site at nucleotide A2058/A2059 of 23S rRNA, mediated by an enzyme encoded by one or more *erm* genes, confers resistance to all MLS_B_ compounds [31, 32]. Mutations at other nucleotides create selective resistance to macrolides, and spares lincosamides [31]. Also, sub-inhibitory concentrations of clindamycin are consistently shown to inhibit *S. aureus* exotoxins [17]. A recent French study demonstrated that sub-inhibitory concentrations of clindamycin did lead to a dramatic decrease in toxin production in various *S. aureus* strains with inducible clindamycin resistance (positive D test) [56]. Hence, it is likely that clindamycin retains its efficacy as a toxin blocker in most *erm*-containing *S. aureus* strains, with the probable exception of those with constitutive resistance.

In conclusion, the proposed CASSETTE study will assess the effect of clindamycin in patients with severe *S. aureus* infections and will provide feasibility for a larger RCT. The study will provide further clinical evidence for such a use.

## Trial status

This is protocol version 2.0 (18 August 2018). Recruitment started 10 July 2018. Expected completion 31 December 2020.

## Additional files

Additional file 1: Renal dose adjustment, adverse events of interest and their grading, and proposed study sites (DOCX 21 kb)

Additional file 2: SPIRIT 2013 Checklist: Recommended items to address in a clinical trial protocol and related documents (DOC 124 kb)

## Data Availability

Not applicable

## Abbreviations

ASID: Australasian Society for Infectious Diseases
CI: Coordinating investigator
CONSORT: Consolidated Standards of Reporting Trials
CRF: Case report form
CRN: Clinical research network
CRP: C-reactive protein
DSMB: Data safety and monitoring board
FBC: Full blood count
HITH: Hospital in the home
HR: Heart rate
HREC: Human research and ethics committee
ICU: Intensive care unit
IV: Intravenous
Menzies: Menzies School of Health Research Global and Tropical Health Division
MIC: Minimum inhibitory concentration
MRSA: Methicillin-resistant *Staphylococcus aureus*
MSSA: Methicillin-susceptible *Staphylococcus aureus*
NHMRC: National Health and Medical Research Council
NOK: Next of kin
PI: Principal Investigator
PO: Per oral
PSI: Protein synthesis inhibitor
PVL: Panton-Valentine leucocidin
QID: Four times a day
RCT: Randomised controlled trials
RR: Respiratory rate
SAB: *Staphylococcus aureus* bacteraemia
SAE: Serious adverse event
SIRS: Systemic inflammatory response syndrome
SPIRIT: Standard Protocol Items Recommendations for Interventional Trials
SSTI: Skin and soft tissue infections
SUSAR: Suspected unexpected serious adverse reaction
TDS: Three times a day
TG: Therapeutic Guidelines (Australia) v15
TGA: Therapeutic goods administration
TSST: Toxic shock syndrome toxin
