# Supplementary material for: CASSETTE—clindamycin adjunctive therapy for severe Staphylococcus aureus treatment evaluation: study protocol for a randomised controlled trial
Source: Trials. 2019 Jun 13;20:353. doi: 10.1186/s13063-019-3452-y (PMC6567404; doi:10.1186/s13063-019-3452-y)
Supplement: Supplementary file 1 — Renal dose adjustment, adverse events of interest and their grading, and proposed study sites (DOCX 21 kb) [file 13063_2019_3452_MOESM1_ESM.docx]

**Renal dose adjustment, adverse events of interest and their grading, and the proposed study sites.**

**Table S1: IV Flucloxacillin dose adjustment for GFR**

| **GFR** | **Adult Dose** | **Paediatric Dose** |
| --- | --- | --- |
| > 50 mL/min | 2g 4-6h | 50mg/kg/dose up to 2g given 4-6h |
| 10 to 50 mL/min | 2g 6h | As above |
| < 10 mL/min | 1g 8h | 50% 4-6h |
| Peritoneal dialysis | 1g 8h | 50% 4-6h |
| Continuous renal replacement therapy | 2g 6h | No dose adjustment necessary |
| Haemodialysis | Not for flucloxacillin (cefazolin 2g 3x/week post dialysis) | 50% 4-6h |

**Table S2: IV Cefazolin dose adjustment for GFR**

| **GFR** | **Adult Dose** | **Paediatric Dose** |
| --- | --- | --- |
| >70 mL/min | 2g 6-8h | 50mg/kg/dose up to 2g given 6-8h |
| 40 to 70 mL/min | 2g 6-8h | 60% 12h |
| 20 to 40 mL/min | 1g 8h | 25% 12h |
| < 20 mL/min | 1g 12h | 10% 24h |
| Peritoneal dialysis | 1g 12h | 25mg/kg/dose 24h |
| Continuous renal replacement therapy | 2g 12 | 25mg/kg/dose 8h |
| Haemodialysis | 2g 3x/week post dialysis | 25mg/kg/dose 24h |

**Table S3: Maintenance dose of IV Vancomycin adjustment for GFR**

| **GFR** | **Starting maintenance dose (Adults)** | **Timing of trough plasma concentration** | **Starting maintenance dose (Paediatrics)** | **Timing of trough plasma concentration** |
| --- | --- | --- | --- | --- |
| > 90 mL/min | 1.5 g 12-hourly | before the fourth dose | 15mg/kg/dose 6-hourly | before the fourth dose |
| 60 to 90 mL/min | 1 g 12-hourly | before the fourth dose | 15mg/kg/dose 12 hourly | before the fourth dose |
| 20 to < 60 mL/min | 1 g 24-hourly | before the third dose | 15mg/kg/dose 24 hourly | before the third dose |
| < 20 mL/min | 1 g 48-hourly | 48 hours after the first dose | 15mg/kg/dose 48-hourly | before the next dose |
| On haemodialysis | 25mg/kg | Immediately prior to next haemodialysis session | 15mg/kg/dose | Immediately prior to next haemodialysis session |

The initial loading dose of 25mg/kg in adults is regardless of GFR value.

The initial loading dose of vancomycin in paediatrics group is at clinician’s discretion.

**Table S4: Adjustment of ongoing IV Vancomycin dose for those on haemodialysis**

| **Vancomycin level (mg/L)** | **Next vancomycin dose (Adults)** | **Next vancomycin dose (Paediatrics)** |
| --- | --- | --- |
| < 5 | 2 g | As per treating clinician |
| 5 - 14 | 1.5 g | As per treating clinician |
| 15 - 20 | 1 g | As per treating clinician |
| 21 - 25 | 0.5 g | As per treating clinician |
| >25 | Not required (check levels prior to the next dialysis session) | As per treating clinician |

*For adjustment after an initial loading dose of 25mg/kg in adults. For subsequent dosing blood is collected for an urgent vancomycin level at the commencement of dialysis. Further dosing of vancomycin will be based on the above table. Administration is timed for the vancomycin infusion to complete simultaneously with the completion of dialysis.*

**Table S5: IV Daptomycin dose adjustment for GFR**

| **GFR** | **Daptomycin dose (Adults)** | **Daptomycin dose (Paediatrics)** |
| --- | --- | --- |
| >50mL/min | 6-12mg/kg q24h | 7-12mg/kg q24h* |
| 11-50 mL/min | 6-8mg/kg q24h | 4 – 6mg/kg q24h |
| 10 mL/min or less but not on haemodialysis | 8mg/kg q48h | 4 – 6mg/kg q48h |
| On continuous renal replacement therapy | 8mg/kg q48h | Clinician directed |
| On haemodialysis | 8mg/kg q48h, dose after dialysis | Clinician directed |

*1 – 6 years old: 12mg/kg/dose Q24h; 7 – 11 years: 9mg/kg/dose Q24H; and 12 – 17 years: 7mg/kg/dose Q24H (Arrieta *et al.* Pediatr Infect Dis J; 2018;37:893–900)

**Table S6: Adverse events of interest and their grading** [Adapted from DAIDS - Table for grading the severity of adult and paediatric adverse events. National Institute of Allergy and Infectious Diseases, Division of AIDS (DAIDS)]

| **Adverse event** | **Grade 1** | **Grade 2** | **Grade 3** | **Grade 4** |
| --- | --- | --- | --- | --- |
| Diarrhoea (age ≥ 1 year) | Transient or intermittent episodes or unformed stools OR Increase of $\leq$3 stools over baseline per 24-hour period | Persistent episodes of unformed to watery stools OR Increase of 4 to 6 stools over baseline per 24-hour period | Increase of ≥7 stools per 24-hour period OR IV fluid replacement indicated | Life-threatening consequences e.g. hypovolemic shock |
| Diarrhoea (age < 1 year) | Liquid stools (more unformed than usual) but usual number of stools | Liquid stools with increased number of stools OR Mild dehydration | Liquid stools with moderate dehydration | Life-threatening consequences e.g. severe dehydration or hypovolemic shock |
| Abdomen pain or cramps | Transient or intermittent AND No or minimal intervention needed | Transient or intermittent AND Needing interventions | Persistent symptoms AND Needing interventions | Potentially life threatening AND needing interventions e.g. peritonism or bowel obstruction |
| Vomiting | Transient or intermittent AND No or minimal interference with oral intake | Frequent episodes with no or mild dehydration | Persistent vomiting resulting in orthostatic hypotension OR Aggressive rehydration indicated | Life-threatening consequences such as hypovolemic shock |
| Rash (describe type) | Localised rash | Diffuse rash OR target lesions | Diffuse rash AND Vesicles or limited number of bullae or superficial ulcerations of mucous membrane limited to one site | Extensive or generalised bullous lesions OR Ulcerations of mucous membrane involving two or more distinct mucosal sites OR Stevens-Johnson syndrome OR Toxic epidermal necrolysis |

**Proposed study sites**

| **Hospital** | **Hospital Type** | **City** | **State** |
| --- | --- | --- | --- |
| Fiona Stanley Hospital | Adult | Perth | WA |
| Royal Perth Hospital | Adult | Perth | WA |
| John Hunter Hospital | Adult | Newcastle | NSW |
| Westmead Hospital | Adult | Sydney | NSW |
| Blacktown Hospital | Adult | Sydney | NSW |
| Royal Melbourne Hospital | Adult | Melbourne | VIC |
| Perth Children’s Hospital | Paediatric | Perth | WA |
| John Hunter Children’s Hospital | Paediatric | Newcastle | NSW |
| Sydney Children’s Hospital | Paediatric | Sydney | NSW |
| Children’s Hospital at Westmead | Paediatric | Sydney | NSW |
| Royal Darwin Hospital | Both | Darwin | NT |
| Townsville Hospital | Both | Townsville | QLD |
